# Microwell culture platform maintains viability and mass of human pancreatic islets

**DOI:** 10.3389/fendo.2022.1015063

**Published:** 2022-11-17

**Authors:** Hiroyuki Kato, Tatsuaki Miwa, Janine Quijano, Leonard Medrano, Jose Ortiz, Akiko Desantis, Keiko Omori, Aya Wada, Kentaro Tatsukoshi, Fouad Kandeel, Yoko Mullen, Hsun Teresa Ku, Hirotake Komatsu

**Affiliations:** ^1^ Department of Translational Research & Cellular Therapeutics, Arthur Riggs Diabetes & Metabolism Research Institute of City of Hope, Duarte, CA, United States; ^2^ AGC Techno Glass, Shizuoka, Japan

**Keywords:** microwells, long-term culture, human pancreatic islets, diabetes, type 1 diabetes, islet transplantation, hypoxia, cell death

## Abstract

**Background:**

Transplantation of the human pancreatic islets is a promising approach for specific types of diabetes to improve glycemic control. Although effective, there are several issues that limit the clinical expansion of this treatment, including difficulty in maintaining the quality and quantity of isolated human islets prior to transplantation. During the culture, we frequently observe the multiple islets fusing together into large constructs, in which hypoxia-induced cell damage significantly reduces their viability and mass. In this study, we introduce the microwell platform optimized for the human islets to prevent unsolicited fusion, thus maintaining their viability and mass in long-term cultures.

**Method:**

Human islets are heterogeneous in size; therefore, two different-sized microwells were prepared in a 35 mm-dish format: 140 µm × 300 µm-microwells for <160 µm-islets and 200 µm × 370 µm-microwells for >160 µm-islets. Human islets (2,000 islet equivalent) were filtered through a 160 µm-mesh to prepare two size categories for subsequent two week-cultures in each microwell dish. Conventional flat-bottomed 35 mm-dishes were used for non-filtered islets (2,000 islet equivalent/2 dishes). Post-cultured islets are collected to combine in each condition (microwells and flat) for the comparisons in viability, islet mass, morphology, function and metabolism. Islets from three donors were independently tested.

**Results:**

The microwell platform prevented islet fusion during culture compared to conventional flat bottom dishes, which improved human islet viability and mass. Islet viability and mass on the microwells were well-maintained and comparable to those in pre-culture, while flat bottom dishes significantly reduced islet viability and mass in two weeks. Morphology assessed by histology, insulin-secreting function and metabolism by oxygen consumption did not exhibit the statistical significance among the three different conditions.

**Conclusion:**

Microwell-bottomed dishes maintained viability and mass of human islets for two weeks, which is significantly improved when compared to the conventional flat-bottomed dishes.

## 1 Introduction

Type 1 diabetes (T1D) is an autoimmune disease in which self-reactive immune cells destroy insulin-producing beta cells (1). Although the exogenous insulin injection has been a standard therapy for patients with T1D, improvement in glycemic control is not satisfactory, leading to secondary complications (2). Beta cell replacement therapy is another promising approach (3, 4); transplantation of the human pancreatic islets isolated from deceased donors has shown that the T1D recipients benefit from improved glycemic control with fewer secondary complications (3, 5–9).

Although islet transplantation is demonstrated to be effective, there are several issues that limit the clinical expansion of this treatment, including donor shortage, the need for lifelong immunosuppression for recipients, and difficulty in maintaining the quality and quantity of isolated islets before transplantation (10). Current clinical islet transplantations are time-sensitive and must be performed within several days after the isolating islets from donors due to the challenge of maintaining the quality and quantity of isolated islets in culture; this critically limits the flexibility of the transplantation schedule, including the selection and preparation of recipients, clinical staff, and pre-transplant quality and safety tests of islets. Therefore, strategies to achieve the extended culture without reducing the quality and quantity of isolated islets could significantly benefit the islet transplantation process for T1D.

One critical factor inducing rapid deterioration of human islets during culture is the hypoxia-induced islet death. Since isolated islets are disconnected from the vascular network in the native pancreas (11–13), the only oxygen (O_2_) source for islets cells is through diffusion from the islet surface, generating the imbalance of O_2_ supply and consumption, leading to cell death in the islet core (14). Islets are metabolically active thus O_2_ consumption is high, which further induces the severe hypoxia (15). The size of isolated islets is inherently determined by their size in the native pancreas between 50 and 500 µm (16), and widely varies across the human donors. We previously demonstrated that cell death in large islets occurs more frequently than in small islets during islet preparations and is correlated to worse transplantation outcomes (17). Islets tend to fuse to create large constructs in the pre-transplant culture period, especially when cultured in crowded conditions, leading to the islet death. Therefore, developing the islet culture method to prevent fusion has excellent potential to improve the islet transplantation strategy.

## 2 Materials and methods

### 2.1 Human islet preparation

All human islet isolations were conducted following standard operating procedures at the Southern California Islet Cell Resource Center at City of Hope, and obtained through the Integrated Islet Distribution Program (18). Three islet batches were tested in this study. Standardized characteristics, consistent with the recommendations of *Diabetes* and *Diabetologia* (19, 20), are summarized in **Supplementary Table 1**, including basic donor information (donor age, donor body mass index, donor HbA1c level, cause of death, and cold ischemia time [time between the cross-clamping for pancreas procurement to pancreas digestion for islet isolation]), islet score and grade (21), and post-isolation islet number.

The following analyses were performed to assess the pre-cultured islets prior to the long-term cultures. Specifically, islet size distribution was assessed at the pre-culture timepoint as described previously (17). Briefly, the size assessments of isolated islets were performed using data collected from viability assay images taken using fluorescein diacetate (FDA; Sigma-Aldrich, Saint Louis, MO, USA) and propidium iodide (PI; Sigma-Aldrich) staining (22). Islet area of all islets captured in the micrographs (IX50, Olympus, Tokyo, Japan) was measured using cellSens imaging software (Olympus). Islet diameter was calculated in which the islets are assumed to be spherical. On average, 106 islets were analyzed for each islet batch (i.e., per isolation). Each islet was categorized by diameter into six size categories (50 – 100, 100 – 150, 150 – 200, 200 – 250, 250 – 300, and >300 µm). The fraction (%) of islets in each size category was calculated by dividing the islet number in each category by the total number of islets in the micrograph. Islet fragments <50 µm were not included in the analysis because of their small impact on the overall volume of the islet preparation.

### 2.2 Microwell dishes

Microwell platforms in a 6 well plate format were tested for seeding isolated human islets: EZSPHERE 901SP-200, EZSPHERE 901SP-300, EZSPHERE 901SP-400, and EZSPHERE 900SP (AGC Techno Glass, Yoshida, Japan). Sizes of the microwells were 220 µm, 300 µm, 370 µm, and 450 µm, respectively (in major axis). The sizes of the microwells were 220 µm, 300 µm, 370 µm, and 450 µm, respectively (major axis). The three-dimensional shape of the microwell was captured using a laser scanning microscope (Keyence, VK-X100, Osaka, Japan) attached with a 20X objective lens (Nikon, OFN25, Tokyo, Japan). The dimension of the microwells was measured as the average of 5 individual wells.

### 2.3 Culture of human islets

**Figure 1:**
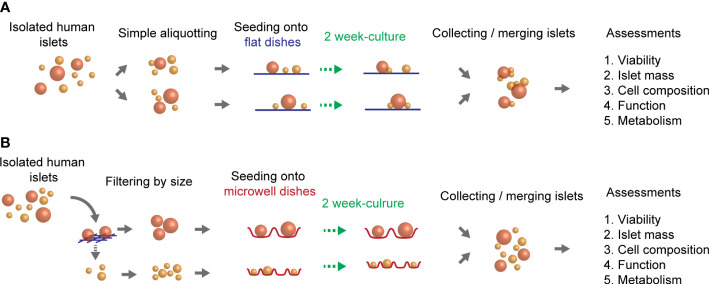
Overview of the culture method of human islets. Isolated human islets were seeded on two different types of culture dishes (flat or microwell) and cultured for two weeks. **(A)** A schematic of culture method using flat-bottomed, conventional dishes. **(B)** A schematic of culture method using microwell-bottomed dishes.

To determine the feasibility of using optimized microwells for a wide range of human isolated islets in the two week-cultures of isolated islets were performed either on flat-bottomed, conventional dishes (**Figure 1A**), or on microwell dishes (**Figure 1B**) in a 6 well-platform or a 35-mm dish format. Approximately 2,000 IEQ of isolated human islets were prepared per group. For the flat-bottom condition, ~2,000 IEQ were simply divided into 2 wells (i.e. ~1000 IEQ/well in a 6-well plate) and cultured. For the microwells, the 160 µm-mesh (Tisch Scientific, Cleves, OH, USA) was used to filter the islets into two groups in different size categories (<160 µm and >160 µm). A small-microwell-dish was used for <160 µm-islets, and a large-microwell-dish was used for >160 µm-islets in the subsequent cultures. Islets were cultured in a CO_2_ incubator at 27°C, with 1 mL per dish of CMRL 1066 culture media (Corning Life Sciences, Tewksbury, MA, USA) supplemented with 0.5% human serum albumin, 0.1 μg/mL insulin-like growth factor-1 (Cell Sciences, Newburyport MA, USA), 10 U/mL heparin sodium (Sagent Pharmaceuticals, Schaumburg, IL, USA), and Penicillin-Streptomycin-Glutamine (Gibco, Waltham, MA, USA). Culture media was replaced every 3 days. Since the aim of this strategy is to improve the overall viability of long-term cultured islets, two week-cultured islets from 2 wells were combined at the end of the culture for the subsequent *in vitro* assessments. Islet mass assessments were performed by the two-dimensional area-based calculation (detailed methods are described in section 5. Islet mass assessments of isolated islets), and the designated islet mass required for each assay was handpicked and confirmed by the area-based calculation.

### 2.4 Viability and purity assessments of isolated islets

Viability assessment of isolated islets was performed using the semi-automated method previously reported (22, 23). Briefly, islets (100 islet equivalent [IEQ]) were stained using FDA and PI, at the final concentration of 0.48 µM and 15 µM in PBS, respectively. Islets were incubated in FDA/PI solution in the dark at 22°C for 5 minutes, followed by washing with PBS and fluorescent imaging on a 96-well plate under a fluorescence microscope (IX50, Olympus). For evaluation, multiple images of a total well were captured and assembled into a single image covering the entire well. FDA-positive area and PI-positive area were mutually exclusive. Therefore, the percent viability of an islet sample was calculated as follows: Overall viability (%) = 100 – (PI-positive area/[FDA-positive area + PI-positive area]) × 100]. Islet purity was assessed by Dithizone (DTZ) staining (iDTZ, Gemini Bio-products, West Sacramento, CA, USA), as previously described (24). Briefly, 50 stained islets were imaged in the bright field (IX50, Olympus) to calculate the purity in the following equation: Purity (%) = (DTZ-stained area/islet area) × 100].

### 2.5 Islet mass assessments of isolated islets

Islet mass was assessed by the two-dimensional area-based calculation using the photomicrographs taken in the bright field. Islets were placed in a 35 mm-dish and imaged (microscope: SZ61, Olympus; digital camera: Infinity2, Teledyne Lumenera, Ottawa, Canada), followed by the area analysis using cellSens imaging software (version 1.12, Olympus). Islet area data was converted to the estimated IEQ; 1 IEQ is defined as an islet with a 150 µm diameter (16). Changes in islet mass during the culture were calculated as relative values to the islet mass on day 0 (pre-culture).

### 2.6. Histological assessment

Isolated islets were fixed in 10% formalin for paraffin embedding and sectioning. For immunofluorescence staining, paraffin sections were subjected to de-paraffinization, re-hydration, antigen retrieval and blocking before antibody incubations. Slides were incubated overnight at 4°C with primary antibodies, followed by the secondary antibodies and DAPI (**Supplementary Table 2**). Images were captured using ZEISS Axio Observer and ZEN lite digital imaging software (Carl Zeiss, Oberkochen, Germany).

To quantify the cell populations within the islets, immunohistochemistry (IHC) staining was performed using the Ventana Discovery Ultra IHC autostainer (Roche Diagnostics, Indianapolis, IN, USA) with primary antibodies and Discovery HQ-HRP-DAB detection system (**Supplementary Table 3**). Images were captured using an IX50 microscope (Olympus) and cellSens software (Olympus). Area-based quantification of IHC-positive cells was performed in each islet (% to islet area); the average % to islet area of ten islets was assessed from a single donor islet preparation (**Supplementary Figure 1**).

### 2.7 Islet function assessment

Glucose-stimulated insulin secretion (GSIS) assay was performed for islet function assessments as described previously (10). Approximately 100 IEQ per well in a 24-well plate cell culture insert (Millipore Sigma, Burlington, MA, USA) were incubated in 1 mL of Krebs-Ringer buffer (KRB) solution containing 2.8 mM glucose for 1 hour, followed by 1 hour incubation in 1 mL of KRB solution containing 28 mM glucose. The buffer was collected after each incubation to measure insulin concentration using a human insulin ELISA kit (Mercodia, Uppsala, Sweden). Insulin secretion was normalized to the IEQ of each sample; islets applied to the 24-well plate cell culture insert were imaged before GSIS to calculate the IEQ using a same manner described in *Islet mass assessments of isolated islets*. The ratio between high insulin secretion and low insulin secretion was used to calculate the stimulation index.

### 2.8 Islet metabolism assessments (OCR)

The O_2_ consumption rate (OCR) assay was performed for islet metabolism assessments (25). Approximately 100 IEQ of islets were plated on a Seahorse XFe islet capture plate (Seahorse Bioscience, North Billerica, MA, USA) and pre-incubated at 37°C in a non-CO_2_-incubator. Measurement of the islet OCR was performed using a Seahorse XFe analyzer (Seahorse Bioscience North Billerica, MA, USA) every 7.5 minutes in the following 4 sequences: 1) baseline at 3 mM glucose for 7 measurements, 2) glucose stimulation at 20 mM for 7 measurements, 3) oligomycin (MilliporeSigma) at 5 µM for 14 measurements, and 4) rotenone (MilliporeSigma) and antimycin (MilliporeSigma) at 5 µM each for 14 measurements. OCR data was normalized by the IEQ applied. Baseline OCR, response to high glucose, ATP dependency and non-ATP respiration dependency were analyzed based on the OCR curves obtained; calculation methods were defined as described in **Supplementary Figure 2**.

### 2.9 Gene expression assays

RNA was isolated from 200 IEQ of islets after the two week-culture (TRI Reagent, Molecular Research Center, Cincinnati, OH, USA, and Direct-zol RNA Microprep, Zymo Research, Irvine, CA, USA). The real-time PCR assays were employed as described previously (26). Relative quantities of each transcript were normalized to an endogenous housekeeping gene (*B2MG*) and expressed as a fold-expression to the CTL (islets cultured on conventional flat dishes). Gene expression data were obtained from three technical replicates for each sample. All primers for *IL1B* (Hs00174097_m1), *TNF* (Hs00174128_m1), *BBC3* (Hs00248075_m1), *FASLG* (Hs00181225_m1), *HMGB1* (Hs001590761_g1) and *B2MG* (Hs00984230_m1) were obtained from Thermo Fisher Scientific (Waltham, MA, USA).

### 2.10 Statistical analysis

Data were reported as the mean ± standard error. Statistical analyses were performed using the JMP 9 program (SAS Institute, Cary, NC, USA). Student’s t-tests were performed for the statistical comparisons between averages of two groups.

## 3 Results

### 3.1 Fate of cultured islets: Fusion of cultured islets exacerbates the intra-islet microenvironment by limiting molecular diffusion

**Figure 2:**
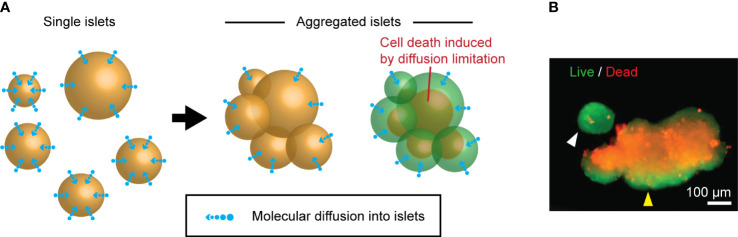
Islet death induced by the fusion during the culture. **(A)** A schematic of molecular diffusion: single islets (left panel) vs. fused islets (right panel). **(B)** A representative live (green color)/dead (red color) staining image of cultured human islets. Single islet (white arrowhead) and fused islets (yellow arrowhead).

Neighboring islets frequently fuse during the culture of isolated human islets. Because the transport/export of molecules within cultured islets solely relies on the passive diffusion through the islet surface, the formation of large, fused islets theoretically limits the diffusion of essential molecules, leading to a suboptimal islet core microenvironment compared to smaller, single islets (**Figure 2A**). Cultured human islets fused with representative islet cell death in the core, while the single islet had minimal cell death (**Figure 2B**). The presence of the uniform thickness of live cells in the outer layer of the islet aggregates is highly suggestive of islet cell survival was diffusion-dependent through the islet surface. These data suggest that maintaining islets in a single unit is critical for improved islet survival during culture.

### 3.2 Size-heterogeneity of the human islets is a challenge in using standardized microwells for islet culture

To prevent the fusion of human islets during the long-term culture, the use of microwell dish could be a potential method to separate individual islets. However, the isolated islets from deceased donors currently used for the clinical islet transplantation are heterogeneous in size, mainly between 50 – 400 µm in diameter, which is inherently determined by the size of islets in the native pancreas. Among the 4 different microwells prepared, the smallest microwell dish (EZSPHERE 901SP-200) was 140 µm (minor axis) × 220 µm (major axis) × 80 µm (depth) in size (**Supplementary Figure 3A**). It accommodates <100 µm-islets, however, seeded islets were unstable and readily floated due to the shallow microwells and large sized islets could not settle within the small microwells. (**Supplementary Figure 3B**). The largest microwell dish prepared (EZSPHERE 900SP) was 330 µm (minor axis) × 450 µm (major axis) × 130 µm (depth) in size (**Supplementary Figure 3C**). It accommodated large, >200 µm-islets; however, multiple small islets occasionally settled in the large microwells and led to unwanted islet fusion (**Supplementary Figure 3D**). These results suggested that using one standardized microwell is not optimal for the culture of heterogeneous-sized islets.

**Figure 3:**
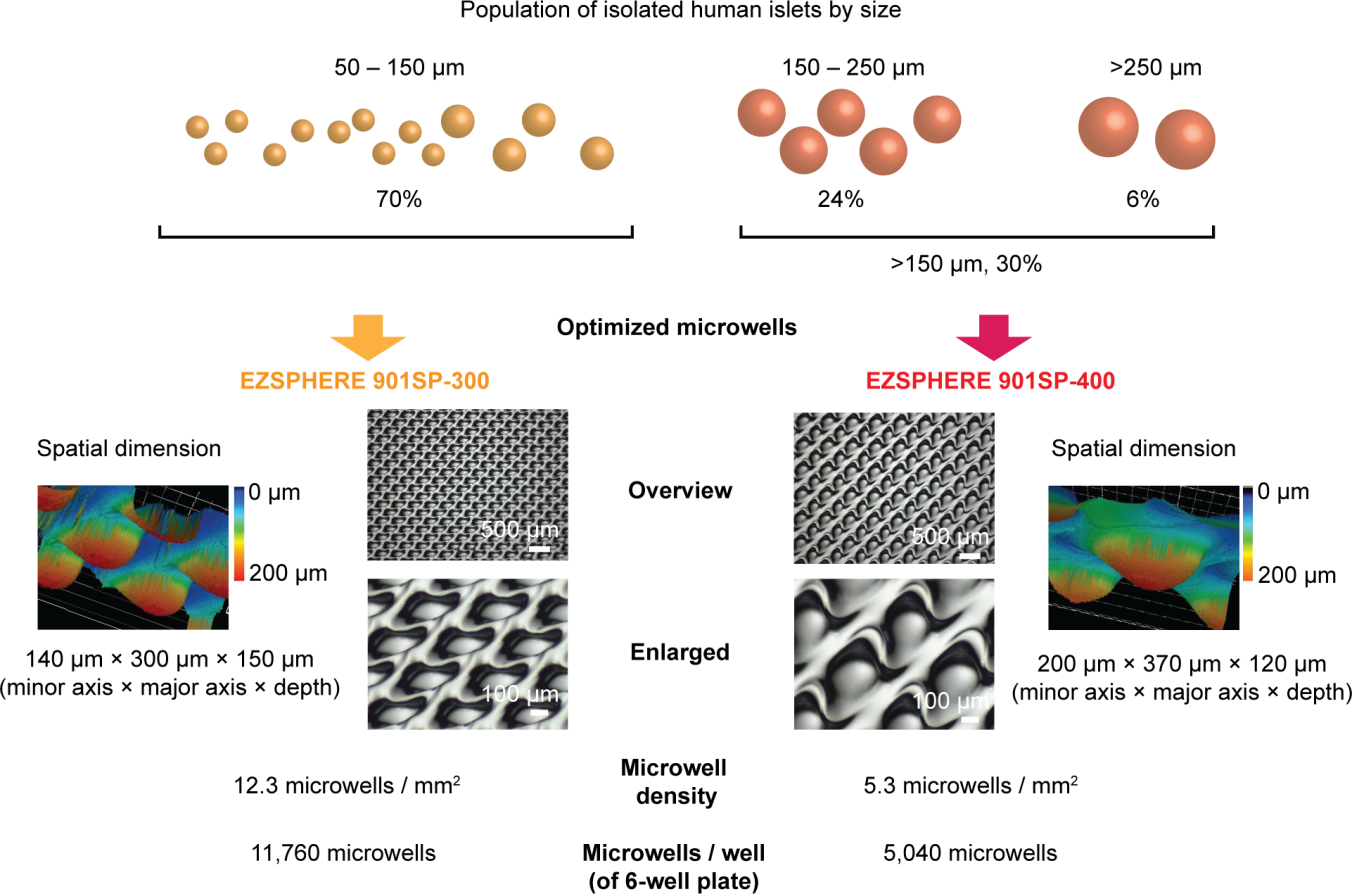
Strategy to culture various-sized human islets using two different-sized microwells. Islet size distribution was obtained from 48 human islet isolations, demonstrated in our previous study; ~70% of islets were 50 – 150 µm in diameter and ~30% in >150 µm. EZSPHERE 901SP-300 (140 µm [minor axis] × 300 µm [major axis]) was used for 50 – 150 µm-islets. EZSPHERE 901SP-400 (200 µm [minor axis] × 370 µm [major axis]) was used for >150 µm-islets. Microscopic laser scanning 3D (inverted image of microwells) and 2D bright-field images are shown.

Theoretically, the preparation of various-sized microwells would be ideal; however, a simple method is preferable and practical for both clinical and experimental settings. Accordingly, we divided isolated islets into two size categories to culture in two different-sized microwells. We used our previously reported data on the size distribution of isolated islets obtained from deceased human donors to determine the optimal cutoff of two size categories (17); on average, 70% of islets fell into 50 – 150 µm, 24% into 150 – 250 µm, and 6% into >250 µm, although islet size had widely varied according to donors. Considering the impact of islet size on islet volume, we set the cutoff at ~150 µm for the following two types of dishes (**Figure 3**). EZSPHERE 901SP-300 has the dimension at 140 µm (minor axis) × 300 µm (major axis) × 150 µm (depth) to accommodate <150 µm-islets, which account for 70% of all islet population. EZSPHERE 901SP-400 has dimension at 200 µm (minor axis) × 370 µm (major axis) × 120 µm (depth) for >150 µm-islets, which account for 30%. In a 6-well plate format, there are 11,760 microwells/well (EZSPHERE 901SP-300) and 5,040 microwells/well (EZSPHERE 901SP-400), with calculated microwell density at 12.3 and 5.3 microwells/mm^2^, respectively. The ratio of microwell density (12.3/5.3) is aligned well with the islet number to be cultured (70%/30%); therefore, a similar percent of microwells can theoretically be occupied by islets in each dish.

### 3.3 Optimal sized microwell platform prevents the fusion of human islets, maintaining viability and mass in long-term culture

Although we chose the optimal-sized microwells for the isolated human islets based on the historically-determined islet size populations (17), islet size can be diverse among different donors and may pose a challenge to culture islets from different donors. To confirm that our microwell sizes can accommodate various islet sizes, we cultured 3 islet batches from 3 different donors with different-sized islet populations (**Supplementary Figure 4**). In the subsequent results, we present the average data of 3 donors as well as the data of individual islet batches.

**Figure 4:**
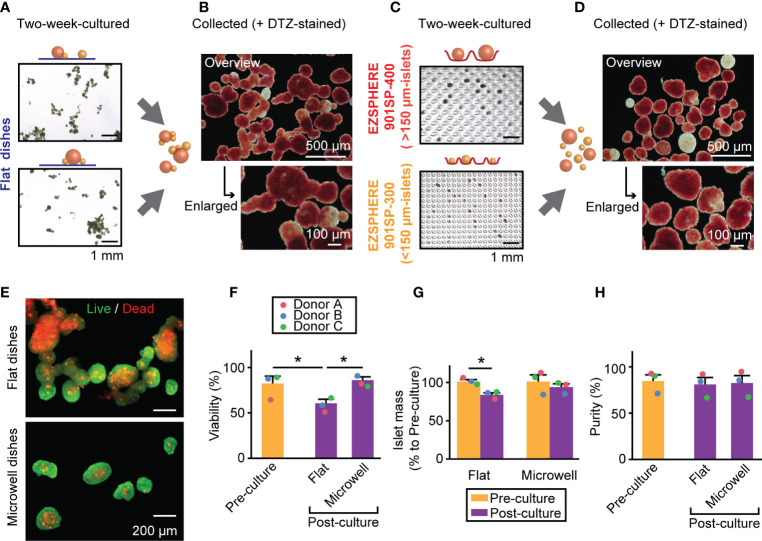
Islet morphology, viability and mass in a two week-culture of human islets. **(A)** A bright-field image of two week-cultured islets on the conventional flat-bottomed dishes. Scale bar: 1 mm. **(B)** Combined islets from two flat dishes and stained with DTZ. Overview (upper, scale bar: 500 µm) and enlarged picture (bottom, scale bar: 100 µm). **(C)** A bright-field image of two week-cultured islets in microwell dishes. Upper picture: >150 µm-islets in EZSPHERE 901SP-400. Bottom picture: 50 – 150 µm-islets in EZSPHERE 901SP-300. Scale bar: 1 mm. **(D)** Combined islets from two microwell dishes, stained with DTZ. Overview (upper, scale bar: 500 µm) and enlarged picture (bottom, scale bar: 100 µm). **(E)** Live/dead staining of two week-cultured islets from flat dishes (upper) and microwell dishes (bottom). **(F)** Viability assessment of islets from three donors. Data of each donor is plotted as different color dots (red, blue and green for Donors A, B and C, respectively). Pre-culture data is presented in yellow bar and post-culture in purple. *P < 0.05 in Student’s t-tests. **(G)** Islet mass analysis shown as % relative value to that of pre-culture. n = 3 donors. *P < 0.05 in Student’s t-tests. **(H)** Islet purity analyzed by DTZ staining. n = 3 donors.

Next, we performed two week-culture of human isolated islets using the method shown in **Figure 1**. **Figure 4A** shows representative images of islets cultured in conventional, flat dishes at two weeks (pre-collected), and DTZ-stained islets demonstrated fusion of multiple islets (**Figure 4B**). However, islets grown in two different-sized microwell dishes (**Figure 4C**) for two weeks exhibited the well-demarcated individual islets with clear borders as single spheroids (**Figure 4D**). Live/Dead staining showed the presence of substantial cell death in the fused-islet constructs retrieved from flat dishes, whereas the cell death is minimized in the non-fused islets retrieved from microwell dishes (**Figure 4E**). Expressions of inflammation-, apoptosis- and necrosis-related genes were investigated to determine the cause of cell death. Inflammation-related gene expressions (*IL1B* and *TNF*) (27, 28) showed the trend of lower inflammation in microwells compared to flat dishes though not statistically significant (**Supplementary Figure 5**). Expressions of apoptosis-related genes (*BBC3* and *FASLG*) (29, 30) and necrosis-related genes (*HMGB1*) (31) in flat dishes and microwells did not demonstrate the primary cause of cell death; rather, they were donor-dependent. Quantification of Live/Dead stained cultured islets demonstrated that the microwell dish significantly improved the viability compared to the flat dish (60.8% vs. 86.2%, *P* = 0.0196; **Figure 4F**). When compared to the viability in pre-culture (82.5%), the post-culture viability on the flat dish was significantly lower (*P* = 0.0352) whereas that on the microwell dish showed no statistical significance (*P* = 0.6670). Subsequently, we assessed islet mass changes between pre-culture and post-culture in both conditions, flat and microwell dishes (**Figure 4G**). Flat dishes significantly reduced islet mass in two weeks (82.7% to pre-culture; *P* = 0.0093), while microwell dishes maintained the pre-culture islet mass (92.8% to pre-culture; *P* = 0.4933). Islet number in each batch can be obtained in **Supplementary Table 4**. Notably, microwell dishes were consistently effective in maintaining islet viability and mass in all islet batches tested, indicating that the microwells can accommodate islet size variations between donors. The two week-culture did not change the islet purity, as measured by the DTZ staining (**Figure 4H**; 85.0% [pre-culture], 81.3% [post-culture, flat dishes], and 82.9% [post-culture, microwell dishes]).

### 3.4 Islet cell composition of key cell types is comparable between the flat dish and microwell dish

**Figure 5:**
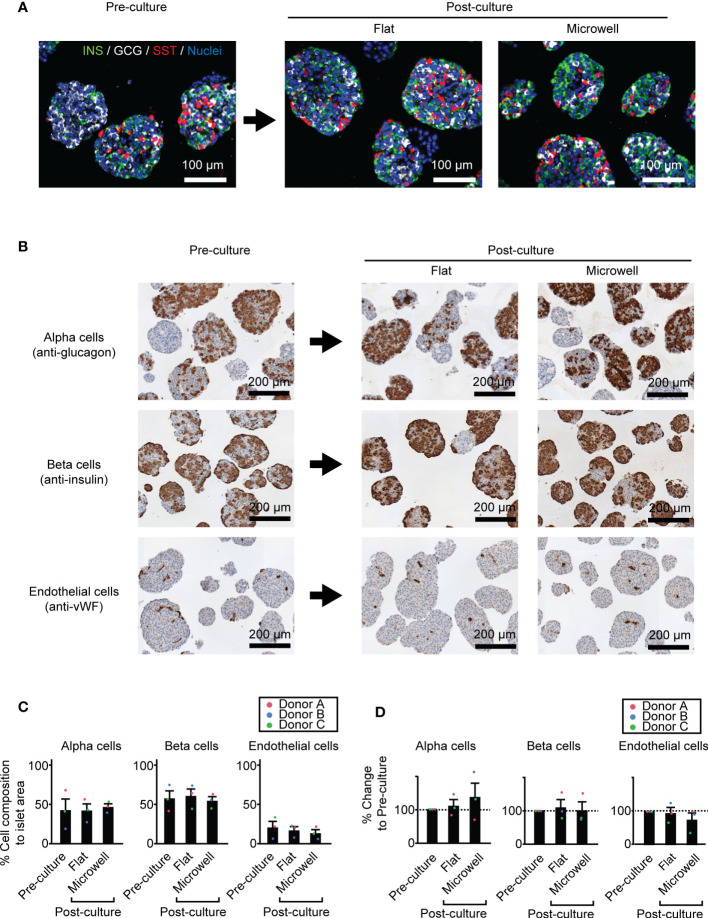
Cell composition of key cell types in two week-cultured human islets. **(A)** Representative immunofluorescence images of pre- and post-cultured (flat dish and microwell dish) islets with major endocrine components (insulin, glucagon and somatostatin. Scale bar: 100 µm. **(B)** Representative immunohistochemistry images of pre- and post-cultured islets for area quantification analyses. Anti-glucagon for alpha cells (upper row), anti-insulin for beta cells (middle row) and anti-vWF for endothelial cells (bottom row). **(C)** The percent (%) of cell composition to islet area pre- and post-culture time points. n = 3 donors. Data of each donor is plotted in different color dots (red, blue and green for Donors A, B and C, respectively). **(D)** Analyses of % changes normalized to pre-culture islets.

**Figure 5A** shows the representative images of pre- and post-cultured (flat dish and microwell dish) islets with major pancreatic endocrine hormones (insulin, glucagon and somatostatin). We observed a similar morphological distribution of these endocrine cell types within the islets. Considering the subsequent transplantation of the long-term cultured islets, we further quantified the potential composition changes of key cell types in the islets during the culture, potentially affecting the transplantation outcome. We chose three key islet cell components, alpha, beta, and endothelial cells, as known determinants on transplantation outcomes (**Figure 5B**) (32–34). Immunohistochemistry staining of these cells demonstrated that islet cell composition of these cell types was comparable among three conditions in pre-culture and post-culture (flat dish and microwell dish) (**Figure 5C**). Further analysis of % changes normalized to pre-culture within the same islet batch showed no statistical difference among three conditions (**Figure 5D**). At the same time, the data of individual islet preparation (Donors A, B and C in **Figures 5C, D**) suggested that cell composition changes are donor-dependent.

### 3.5 Human islets cultured on the flat dish and microwell dish demonstrate comparable function and metabolism

**Figure 6:**
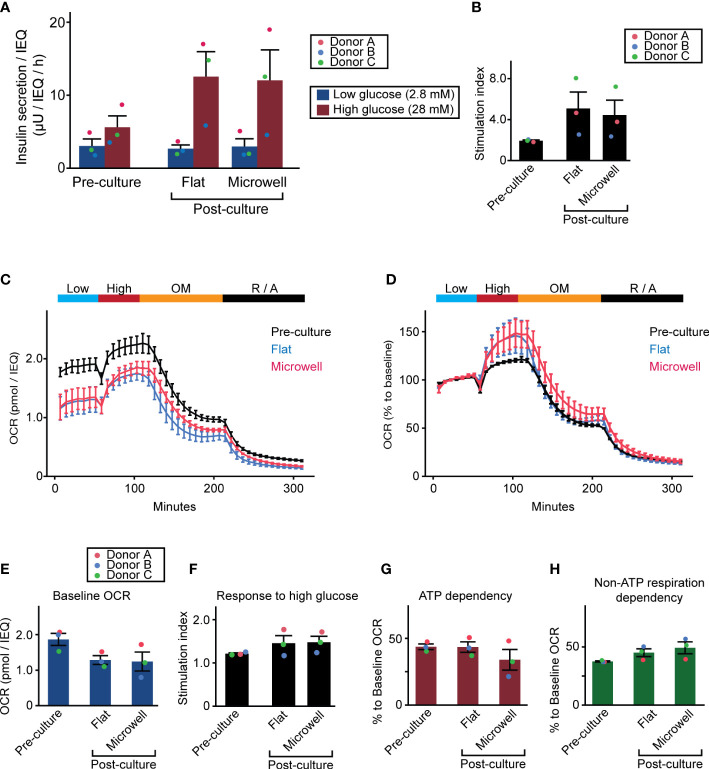
Insulin-secreting function and metabolism of two week-cultured human islets. **(A)** Glucose-stimulated insulin secretion assay was performed with 1 hour of low glucose exposure followed by 1 hour of high glucose exposure. Insulin secretion was normalized by the islet number (IEQ) applied. Data of pre- and post-cultured (flat dish and microwell dishes) islets are presented. n = 3 donors. Data of each donor is plotted in different color dots (red, blue and green for Donors A, B and C, respectively). **(B)** Stimulation index calculated as the ratio of high insulin secretion over low insulin secretion. **(C)** OCR assay with 4 solution phases with 1) baseline at 3 mM glucose, 2) glucose stimulation at 20 mM, 3) 5 µM oligomycin, and 4) 5 µM rotenone and 5 µM antimycin. n = 3 donors. Absolute OCR data was normalized by the islet number (IEQ) applied. Data plots are the average of 3 donors. Data of individual donor islets is available in **Supplementary Figure 6**. **(D)** OCR assay data analysis normalized to the baseline OCR. **(E–H)** Analyses of baseline OCR, response to high glucose, ATP dependency, and non-ATP respiration dependency in pre- and post-cultured (flat dish and microwell dishes) islets. Detailed method for the analysis is described in **Supplementary Figure 2**. Data of each donor is plotted in different color dots (red, blue and green for Donor A, B and C, respectively).

Because a major concern of human islets during long-term culture is functional capacity prior to transplantation, we evaluated next whether microwell dish affects the insulin secretion and metabolic function of islets. First, we evaluated the glucose-stimulated insulin-secreting function of islets (**Figure 6A**). Post-cultured islets on the microwell dish and flat dish exhibited comparable function, while insulin secretion widely varied according to the donors. Post-cultured islets were well responsive to high glucose, although they were not statistically significant when compared to the pre-cultured islets (**Figure 6B**). Next, we assessed OCR for islet metabolism, including basal respiration, glucose-response, ATP dependency and non-ATP dependency (see **Supplementary Figure 2** for assessment method). Post-cultured islets cultured in flat dish and microwell dish groups from the three donors showed similar OCR curves and was overall lower than pre-cultured islets (**Figure 6C**). Normalized data to the baseline OCR is presented in **Figure 6D**. Analysis of absolute OCR value in the baseline showed a trend of reduced basal metabolism in post-cultured islets to that in pr e-cultured islets (but no statistical significance; **Figure 6E**). Detailed metabolic analyses of glucose-induced OCR response (**Figure 6F**), ATP dependency of the islet metabolism (**Figure 6G**), and non-ATP dependency of the islet metabolism (**Figure 6H**) demonstrated no statistical significance among 3 groups (pre-cultured, post-cultured in the flat dish and post-cultured in the microwell dish); however, results showed trends of increased OCR response to the high glucose and increased non-ATP respiration dependency in post-cultured islets compared to the pre-cultured islets. OCR was highly donor dependent. Individual data is available in **Supplementary Figure 6**. Overall, these data suggests that the microwell dish does not significantly affect islet function and metabolism during long-term culture compared to the flat dish.

## 4 Discussions

In this study, we demonstrated the advantage of using a microwell platform over the conventional flat-bottomed dishes to culture human isolated islets. The main goal of the microwell culture system was to prevent the fusion of islets that frequently occurs in the conventional culture system, thereby mitigating the deleterious islet microenvironment observed in large fused cultured islets. The microwell platform thus improved islet viability and increased islet mass without sacrificing islet purity and function. Because a wide range of the size of human islets poses a challenge for using the standardized microwell format, we designed the optimal-sized microwells to accommodate various islet sizes.

The microwell culture method could significantly contribute to the current clinical islet transplantation preparations and improve transplantation outcomes and process. Islet mass and viability are the critical determinants of islet transplantation success (7, 23, 35); thus, culturing islets in microwells is clinically impactful since this culturing platform maintains isolated islet mass and viability in the pre-transplantation period. Importantly, this platform is easily adaptable with minimal modification to the current islet cultures; a conventional strainer separates isolated islets into two different-sized populations, which will be subsequently cultured in two different-sized microwells.

Islets can be maintained in the microwell platform in the long term, and achieving the long-term islet culture can be a potential strategy for any clinical islet transplantation. For example, current islet transplantations are performed on a tight schedule, a few days after the isolation of islets from donors due to the rapid deterioration of islet viability with the current pre-transplantation culturing method. Extended pre-transplantation time would give the recipients and clinicians more flexibility to prepare the transplantations with better logistics and safety. Additionally, the long-term islet culture may pave the way for using islets from multiple donors in a single transplantation. In the current practice, the low yield of isolated islets from a single donor is the frequent cause for not going forward with clinical islet transplantation. Since islet number is a critical factor in the transplant outcome (7, 35), combining the multiple donors’ islets could be a potential approach with careful consideration of immune response issues. Another potential clinical transplantation strategy for diabetic patients is the transplantation of long-term cultured islets after kidney transplantation to treat renal failure caused by diabetic glucotoxicity in the diabetes patients (36–38). Because kidney transplantation is more invasive and induces intense immune responses and cytokine storms (39), delayed islet transplantation after the initial cytokine surge has subsided would be optimal. This strategy allows us to use the same donor for islets and kidney.

Furthermore, in the preclinical research setting, rapid islet deterioration, including reduced viability and volume loss, is a major limitation, especially when the research requires long-term tracking of investigational agents. The use of microwells minimizes the islet deterioration, and investigators can focus on their biological interests in the long term.

Previous publications demonstrated that pseudo-islets remodeled by dispersion and subsequent reaggregation from the isolated islets could be more uniform in size with improved function compared to the isolated islets (40, 41). Although interesting, pseudo-islets exhibited cell composition changes in the extracellular matrix and endothelial cells (42). More importantly, the loss of cell mass during the remodeling procedures is often overlooked. Because the islet mass is a critical factor for the success of clinical islet transplantation for diabetes patients (35), loss of the islet mass could be a critical flaw. Thus, using isolated islets without dispersion-reaggregation could be advantageous than using pseudo-islets.

Microwell platforms have been used to generate cell aggregates from single cells (43–45), in which the size of the microwell and cell seeding density determine the size of cell aggregates. In this study, we used microwells for culturing islets isolated from deceased donors with inherently determined islet sizes. Microwells separate the individual islets to prevent islet fusion and maintain islet viability and mass; however, appropriate microwell size is critical. Previous literature demonstrated that microwells ~400 µm in diameter maintained functional human islets for 7 days (46). Another study showed that 500 µm-microwells effectively segregated islets for the short-term, with a shaking culture condition mimicking islet shipping (47); however, multiple islets in a well were observed, which indicates the importance of islet-size-specific microwells. We addressed the issue by preparing different-sized microwells, minimizing the fusion of large islets with a simple and practical approach.

One challenge in the strategy of our cutoff at 150 µm could be the biological variation in islet sizes among donors. We validated the feasibility of this cutoff strategy using islets from three different human donors. Although these islet preparations showed different islet size distributions, our microwell strategy successfully improved the viability and islet mass recovery in all donor cases compared to the conventional flat-bottomed flasks, demonstrating the potential clinical applicability of this platform.

One important factor which affects transplantation outcomes is the size of isolated islets; smaller isolated pancreatic islets are more functional than larger islets leading to improved transplantation outcomes (17, 48–50). Because our strategy requires the separation of small and large islets with a cutoff at 150 µm, the ratio of small and large islet numbers can be counted which potentially predicts successful transplantations.

Although the microwell strategy prevented islet fusion and improved viability and mass recovery of cultured islets over the conventional flat bottom dish culture, they did not demonstrate the significant changes in function and metabolism evaluated by GSIS and OCR, suggesting that the remnant live islet cells in the conventional, flat-dish culture function properly. Furthermore, we saw a trend toward improved insulin secretion and OCR stimulated in the high glucose condition cultured for two weeks compared to those in pre-culture islets. The potential reason is that the pre-culture islets did not fully recover from the damage that occurred in the isolation procedures (i.e. digestion process). Histologically, two week-culture did not alter the population of major endocrine cells within the islets on average (among 3 donor-islet preparations) in contrast to previous studies, which reported reduced endocrine cells after extended culture (51, 52). However, cell composition changes were highly donor-dependent, and there were cases with increased alpha cells and decreased beta cell populations, which is not ideal in beta cell replacement therapies. Further assessments of islets cultured in the long-term are required, including transplantations into diabetic animals.

Intra-islet endothelial cells play a critical role in effective islet engraftment after transplantation (32–34). Although islets in the native pancreas are highly vascularized (33, 53), intra-islet endothelial cells rapidly decrease after islet isolation and subsequent culture (54). We also observed one case with drastically reduced endothelial cell composition in long-term cultured islets. Providing a more physiological environment in addition to microwell culture could potentially ameliorate the quality of long-term cultured islets; for example, a dynamic culture system mimicking tissue fluid movement can enhance molecular diffusion and stimulate endothelial cells with the maintenance of appropriate shear stress (55).

Collectively, our microwell culture strategy optimized for isolated human islets has significant potential to enhance the current human islet transplantation outcomes by improving the viability and mass of cultured islets. Use of microwell dishes can also benefit experimental research by achieving longitudinal islet observation.

## Data availability statement

The original contributions presented in the study are included in the article/**Supplementary Material**. Further inquiries can be directed to the corresponding author.

## Author contributions

Conceptualization: TM and HKo. Methodology: TM, AW, KT, HTK, and HKo. Investigation: TM, HKa, JQ, LM, JO, AD, and KO. Visualization: TM, HKa, JO, and HKo. Supervision: HKo. Writing—original draft: TM, HKa, and HKo. Writing—review & editing: HKa, JO, FK, YM, HTK, and HKo. All authors contributed to the article and approved the submitted version.

## Funding

This study was supported in part by grants from the Nora Eccles Treadwell Foundation, National Institutes of Health, R03DK129958-01, and the Juvenile Diabetes Foundation, 3-SRA-2021-1073-S-B to HKo.

## Acknowledgments

We acknowledge the manufacturing team at the Southern California Islet Cell Resources Center, Arthur Riggs Diabetes & Metabolism Research Institute of City of Hope, for preparing the isolated human islets. Research reported in this publication included work performed in the Pathology Core supported by the National Cancer Institute of the National Institutes of Health under grant number P30CA033572. The content is solely the responsibility of the authors and does not necessarily represent the official views of the National Institutes of Health. We thank Sung Hee Kil, Ph.D. for critical reading and editing of the manuscript.

## Conflict of interest

This study was performed as a collaborative study between AGC Techno Glass and Arthur Riggs Diabetes & Metabolism Research Institute of City of Hope. TM, AW, and KT are the employees at AGC Techno Glass.

The remaining authors declare that the research was conducted in the absence of any commercial or financial relationships that could be construed as a potential conflict of interest.

## Publisher’s note

All claims expressed in this article are solely those of the authors and do not necessarily represent those of their affiliated organizations, or those of the publisher, the editors and the reviewers. Any product that may be evaluated in this article, or claim that may be made by its manufacturer, is not guaranteed or endorsed by the publisher.
