# Supplementary material for: Microwell culture platform maintains viability and mass of human pancreatic islets
Source: Front Endocrinol (Lausanne). 2022 Nov 17;13:1015063. doi: 10.3389/fendo.2022.1015063 (PMC9712283; doi:10.3389/fendo.2022.1015063)
Supplement: Supplementary file 5 [file DataSheet_1.pdf]

# *Supplementary Material*

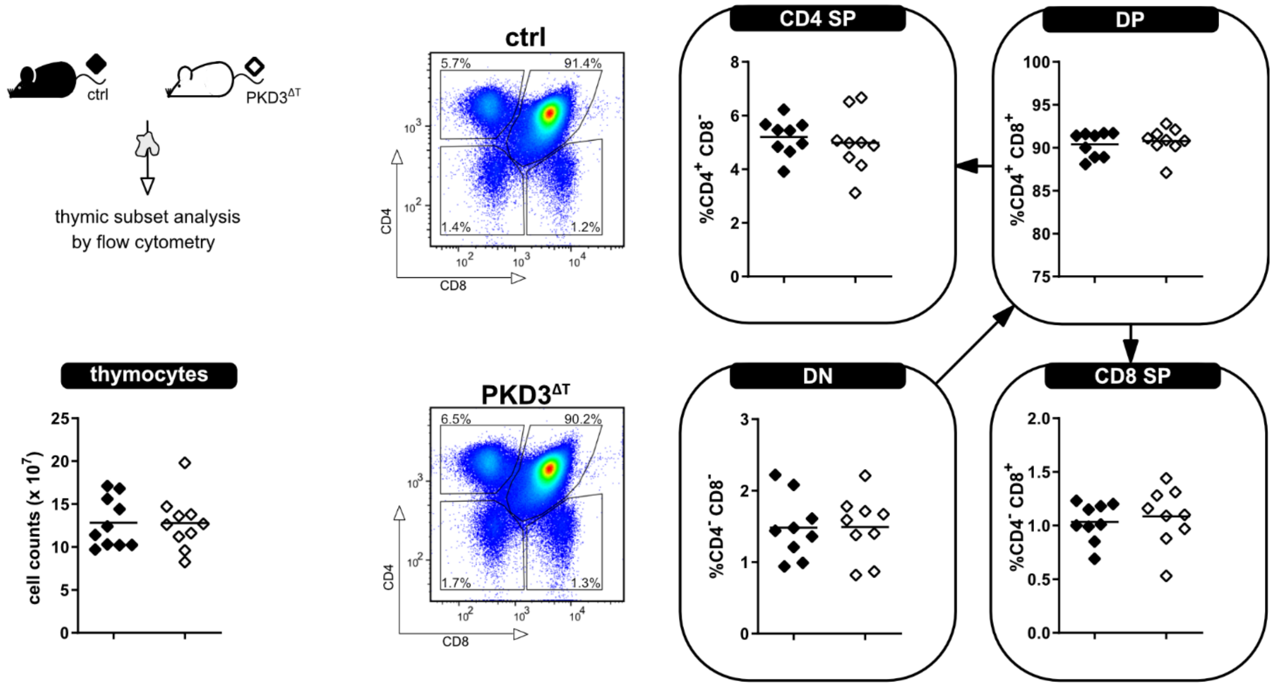


**Supplementary Figure 1. Unaltered thymic development of PKD3$^{\Delta T}$ mice**

Thymocytes from either PKD3$^{\Delta T}$ mice (◇) and respective controls (◆) were counted and analyzed for CD4 and CD8 expression by flow cytometry. Results are shown with a representative dot plot together with summarizing graphs showing relative proportions of CD4/8 double negative (DN), double positive (DP) and CD4 as well as CD8 single positive (SP) cells. (n = 9; from 3 independent experiments)
