# Supplementary material for: Microwell culture platform maintains viability and mass of human pancreatic islets
Source: Front Endocrinol (Lausanne). 2022 Nov 17;13:1015063. doi: 10.3389/fendo.2022.1015063 (PMC9712283; doi:10.3389/fendo.2022.1015063)
Supplement: Supplementary file 6 [file DataSheet_2.pdf]

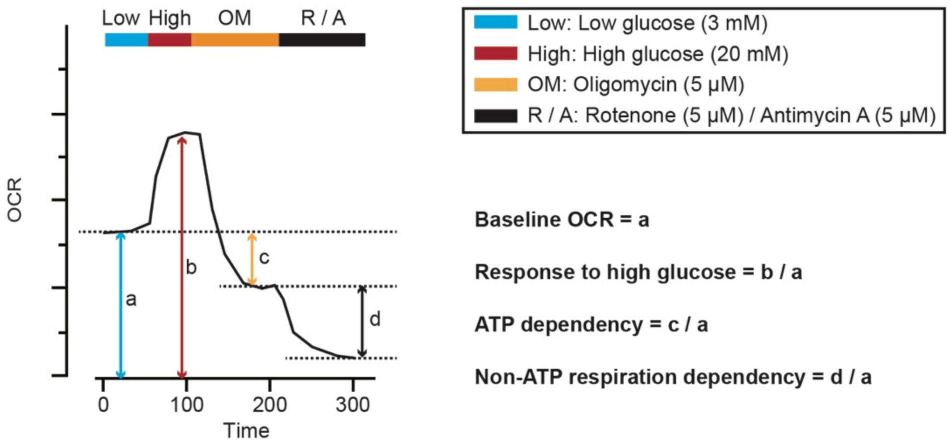

Supplementary Figure 2. Method of the islet metabolism. Islet metabolism was evaluated using oxygen consumption rate (OCR) assay, with sequential administration of 3 mM glucose (basal media), 10 mM glucose, 5 µM oligomycin in basal media, and 5 µM rotenone / 5 µM antimycin in basal media. A schematic shows the calculation method for baseline OCR, response to high glucose, ATP dependency and non-ATP respiration dependency.
