# Supplementary material for: Microwell culture platform maintains viability and mass of human pancreatic islets
Source: Front Endocrinol (Lausanne). 2022 Nov 17;13:1015063. doi: 10.3389/fendo.2022.1015063 (PMC9712283; doi:10.3389/fendo.2022.1015063)
Supplement: Supplementary file 7 [file DataSheet_3.pdf]

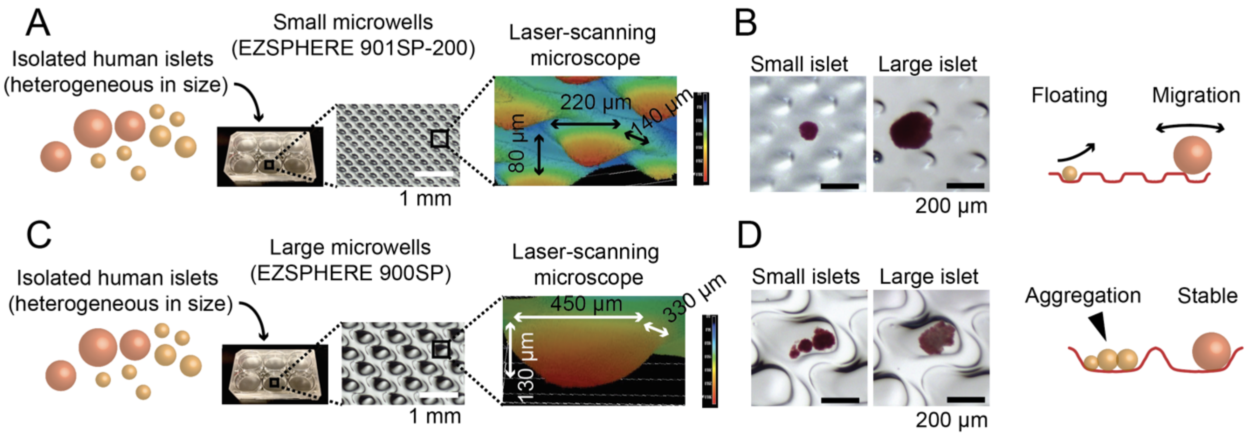

Supplementary Figure 3. Challenge using standardized microwells in islet culture due to their heterogeneous sizes. (A) Appearance of the small-sized microwells **(**EZSPHERE 901SP-200) in bright field (left panel) and laser-scanning microscope image (right panel). **(B)** Microphotographs of small (50 – 100 µm) and large (>200 µm) islets in the small-sized microwells captured with a bright field microscope (left panel). A schematic of different-sized islets seeded in the small-sized microwells (right panel). **(C)** Appearance of the large-sized microwells **(**EZSPHERE 900SP) in bright field (left panel) and laser-scanning microscope image (right panel). **(D)** Microphotographs of small (50 – 100 µm) and large (>200 µm) islets in the large-sized microwells captured with a bright field microscope (left panel). A schematic of different-sized islets seeded in the large-sized microwells (right panel).
