# Supplementary material for: Microwell culture platform maintains viability and mass of human pancreatic islets
Source: Front Endocrinol (Lausanne). 2022 Nov 17;13:1015063. doi: 10.3389/fendo.2022.1015063 (PMC9712283; doi:10.3389/fendo.2022.1015063)
Supplement: Supplementary file 8 [file DataSheet_4.pdf]

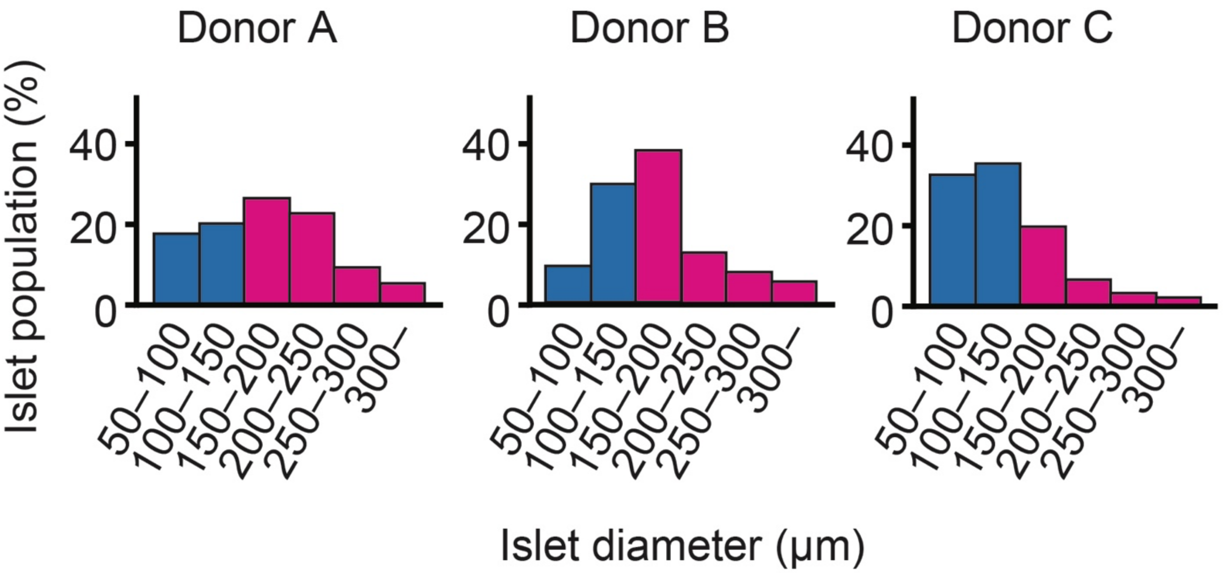

Supplementary Figure 4. Islet size distribution of three donors. Islet size distribution was assessed in each donor (Donors A, B and C). On average, 106 islets were analyzed (Donor A:80, Donor B:130 and Donor C:108). Fraction (%) of islets in each size category were calculated. Blue and pink bars show the islet population below and above 150 μm in diameter, respectively.
