# Supplementary material for: Microwell culture platform maintains viability and mass of human pancreatic islets
Source: Front Endocrinol (Lausanne). 2022 Nov 17;13:1015063. doi: 10.3389/fendo.2022.1015063 (PMC9712283; doi:10.3389/fendo.2022.1015063)
Supplement: Supplementary file 9 [file DataSheet_5.pdf]

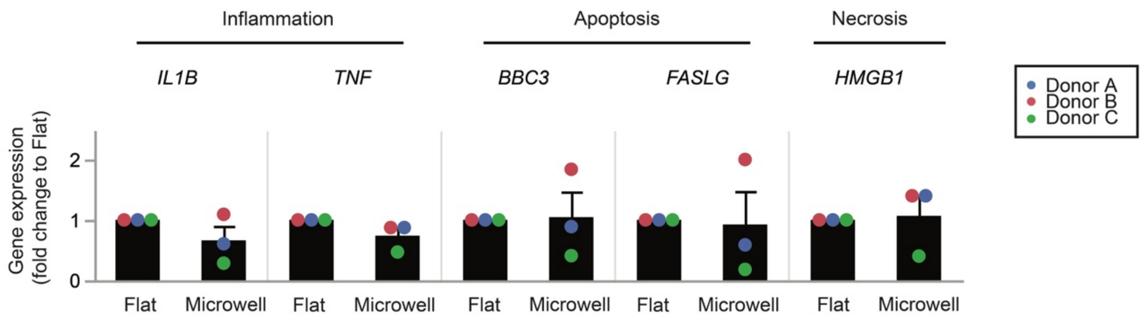

Supplementary Figure 5. Gene expression assays of two week-cultured islets from flat dishes and microwell dishes. Data of each donor is plotted as different color dots. Gene expressions of target genes were expressed as fold changes to those of islets from flat dishes. No statistical significance was seen between flat vs. microwell among 5 genes tested.
