# Supplementary material for: Microwell culture platform maintains viability and mass of human pancreatic islets
Source: Front Endocrinol (Lausanne). 2022 Nov 17;13:1015063. doi: 10.3389/fendo.2022.1015063 (PMC9712283; doi:10.3389/fendo.2022.1015063)
Supplement: Supplementary file 10 [file DataSheet_6.pdf]

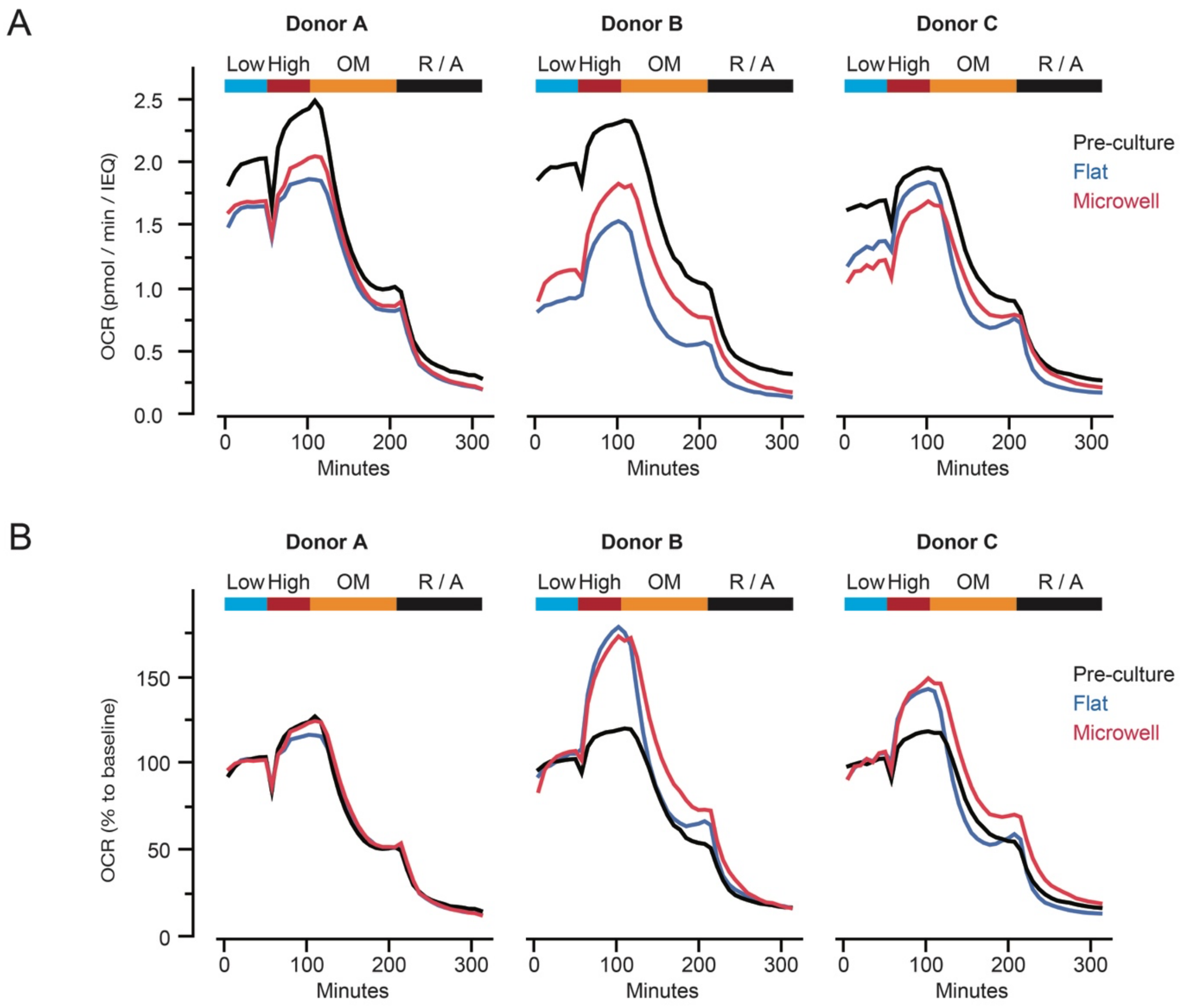

Supplementary Figure 6. OCR assay data of individual donor islets. **(A)** Absolute OCR data normalized by the islet number (IEQ). **(B)** OCR assay data analysis normalized to the baseline OCR.
